# Laparoscopic vs open Ladd’s procedure for malrotation in neonates and infants: a propensity score matching analysis

**DOI:** 10.1186/s12893-022-01487-1

**Published:** 2022-01-26

**Authors:** Weike Xie, Zhongwen Li, Qi Wang, Lei Wang, Yongkang Pan, Chaoxiang Lu

**Affiliations:** Department of Neonatal Surgery, Xi’an Children Hospital, 69 Xiyuyuanxiang, Xi’an, 710003 Shaanxi China

**Keywords:** Laparoscopy, Intestinal malrotation, Infants, Ladd’s procedure, Therapy

## Abstract

**Background:**

Laparoscopic Ladd’s procedure for malrotation in children is still a controversial approach. Although some retrospective studies have compared the outcomes of the two types of procedure with inconsistency outcomes. Currently, there are few large-scale studies on laparoscopic treatment in malrotation with neonates and infants. We did a study based on propensity score matching to compare the effects of the two kinds of approach in neonates and infants. To investigate the therapeutic effect of laparoscopy and open Ladd’s procedure by the propensity score-matching (PSM) to enhance the validity of the comparison.

**Methods:**

A total series of 143 cases of intestinal malrotation without intestinal necrosis was included in the study during the 8 years from January 2012 to January 2020, including 68 cases of open Ladd’s procedure and 75 cases of laparoscopic Ladd’s procedure including five cases of transfer laparotomy. By a propensity score 1:1 matching, 62 patients were stratified for each group.

**Results and conclusion:**

There was no significant difference in volvulus degree, weight and gender between the two groups (p > 0.05). Laparoscopic surgery took more time than open surgery (105.9 min vs 70.6 min, p < 0.05), but it had less hospital stay (12.4 days vs 14.6 days, p < 0.05) or less incision infection (0 vs 6, p < 0.05). There was no significant difference between the two groups at the time of first defecation, blood loss, time of full feeding and reoperation (p > 0.05). The cosmetic effect of laparoscopic surgery is better than that of open surgery. Laparoscopic Ladd’s procedure is a safe approach. It can reduce the length of hospital stay and incision infection, but the operation time was extended, the other complications are similar compared with open procedure for intestinal malrotation in neonates and infants.

## Introduction

Intestinal malrotation is the result of abnormal intestinal rotation and fixation during the fetus. It is usually characterized by biliary vomiting and intestine volvulus, which can lead to intestinal necrosis. Open Ladd’s procedure is the standard treatment of intestinal malrotation. The use of laparoscopy in intestinal malrotation in infants has been gradually developed since 1995 [1]. However, laparoscopic Ladd’s procedure for malrotation in children is still a controversial approach [2]. The patient’s age and volvulus may lead to a different conclusion. It may mainly be due to the high proportion of transfer and high risk of redo [3, 4]. Although some retrospective studies have compared the outcomes of the two procedures with inconsistency outcomes, but there are few reports of laparoscopic surgery in neonates and infants. We did a study based on propensity score-matching analysis to compare the effects of the two procedures. The purpose of this study was to investigate the efficacy and safety of laparoscopic treatment of intestinal malrotation compared with open procedure in neonates and infants by a propensity score matching cohort study.

## Methods

### Patient’s selection

A retrospective analysis was made in our center from January 2012 to January 2020. The study was approved by the Xi’an Children hospital ethics committee and met the guidelines of the Helsinki declaration. Informed consent of parents or legal guardians is obtained before operation and study. The inclusion criteria are as follows: Infant diagnosed as intestinal malrotation without intestinal necrosis. Exclusion criteria: Infants with malrotation of the intestine and other simultaneous malformations that require treatment.

### Surgical technique

#### Laparoscopic Ladd’s procedure

**Fig. 1:**
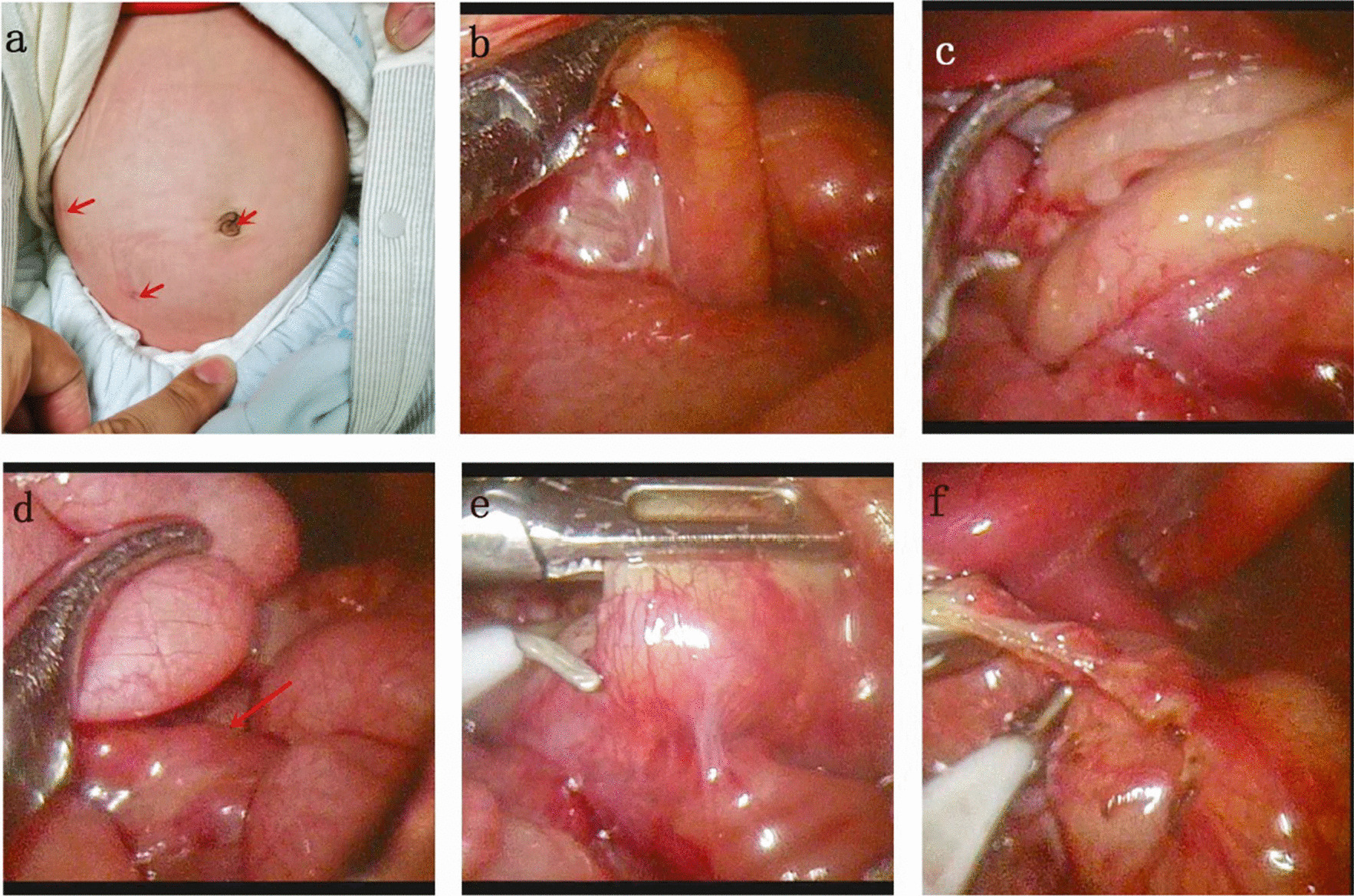
**a** The arrows point to the locations of the three trocars. **b** The ascending colon penetrates from the left side of the mesentery to the rear, suggesting a clockwise volvulus. **c** When the colon is reduced, the transverse colon and ascending colon are fully exposed. **d** It is necessary to explore the jejunum from the ileum because part of the mesentery may have folded. **e** After complete reduction, the Ladd band was cut. **f** Cut the greater omentum from the transverse colon

The procedure is briefly illustrated in Fig. 1. The child was placed in a supine position, and the trocar was placed through the umbilicus, right upper abdomen and right lower abdomen, and the pneumoperitoneum pressure was 6–8 mmHg. As showing in Fig. 1a. After the pneumoperitoneum was established, we explored the cecum from left to right from the transverse colonic and splenic region. If the cecum was drilled into the back of the intestine, it was suggested that the cecum was twisted counterclockwise, as shown in Fig. 1b. The bowel can be re positioned by pulling the root of the bowel from the left abdomen to the right. When the transverse and ascending colons were presented as letter “C”, the reduction of colon torsion was completed in (Fig. 1c).Then from the cecum to the jejunum retrograde probe. At this time, we found that some children had folded or partially twisted mesentery (Fig. 1d). After complete reduction of intestinal torsion, the Ladd’s band was released (Fig. 1e). In order to fully expand the mesentery, the greater omentum was released from the transverse colon (Fig. 1f). At our center, the appendix is removed or not at the parents’ option.

#### Open Ladd’s procedure

The infants was placed in a supine position, and the right upper abdomen is made into the abdomen through a transverse incision. If the small intestine is torsion, the intestine is reset, usually counterclockwise. Ladd’s band is cut to make the duodenum fall vertically along the right side of the spine. After appendectomy, the small intestine is placed on the right side of the abdomen, and the colon is placed on the left of the abdomen and then close the abdominal incision.

### Postoperative treatment and follow-up

When gastric tube drainage fluid becomes light green, oral milk was given, and the feeding amount reaches enough, the patient will be discharged from the hospital. If the incision infection occurs, the incision is drained, and then the dressing is changed daily until the incision is healed. The follow-up period was 6 months to 8 years.

The general condition of the child was recorded including age, sex and weight. Operation time, volvulus degree, first defecation after surgery, full feeding time, incision, length of stay, redo were also recorded.

### Statistical analysis

The patients baseline characteristics were expressed as the mean ± standard deviation or median (range) for continuous data. Comparison of categorical variables was performed using the χ^2^ test or Fisher’s exact test with Yates correction when appropriate. Unpaired Student *t* test was used to compare differences in continuous parametric variables and the Mann–Whitney U test for continuous nonparametric variables when appropriate. In order to compare perioperative out comes of laparoscopic and open surgery for malrotation, we performed a propensity score-matching analysis. Propensity scores were calculated by bivariate logistic regression, including the following variables that might be considered as potential baseline confounders between the groups: age, sex, weight and volvulus degree. We matched propensity scores 1:1 with the use of the nearest neighbor methods without replacement by using a 0.02 calipers width to achieve the goal of minimizing differences between groups. Significance was defined as a p value of less than 0.05. The statistical analysis was performed using the SPSS version 26.0 (SPSS, Inc., Chicago, IL).

## Results

A total of 143 children underwent intestine malrotation without intestinal necrosis, excluding five children who underwent laparoscopy transfer to laparotomy. According to the wishes of the parents, the children were divided into two groups, including 68 cases of open laparotomy and 75 cases of laparoscopic Ladd’s procedure. By Propensity score-matching (PSM) to enhance the validity of the comparison, 62 patients were included in each group. No difference in blood loss, volvulus degree, body weight and sex ratio between the two groups (Table 1). The operation time of the children in the laparoscopic group was 106.6 ± 36.7 min, and that of the open operation group was 72.1 ± 16.8 min before PSM. The operation time of the laparoscopic group was longer than that of the open but it had less hospital stay (12.4 vs 14.6, p < 0.05) or less incision infection (0 vs 6, p < 0.05). Postoperative results and complications were found, and the time of the first defecation, full feeding, and the time of the second operation were all similar (Table 2). The cosmetic effect of laparoscopic surgery is better than that of open surgery. Of the children in transit, the reasons for transfer were bleeding in two cases and at the beginning of the learning curve in three cases.

**Table 1:** Baseline of the open and laparoscopy Ladd’s procedure

	Before PSM			After PSM		
	Open (n = 68)	Laparoscopy (n = 75)	p	Open (n = 62)	Laparoscopy (n = 62)	p
Weight (kg)	3.2 ± 0.6	3.3 ± 0.9	0.437	3.2 ± 0.7	3.2 ± 0.7	0.94
Gender (M/F)	46/22	52/23	0.828	40/20	40/20	1.000
Age (d)	18.9 ± 19.6	24.7 ± 26.1	0.134	18.9 ± 19.8	18.6 ± 17.8	0.775
Volvulus degree	346.9 ± 197.5	369.8 ± 182.7	0.388	346.9 ± 197.5	360 ± 181.5	0.654
Transfer (Y/N)	–	5	–	–	4	–

**Table 2:** Recovery and complications baseline of the open and laparoscopy Ladd’s procedure

	Before PSM		*p*	After PSM		*p*
	Open (n = 68)	Laparoscopy (n = 75)		Open (n = 62)	Laparoscopy (n = 62)	
Bleeding (ml)	7.6 ± 8.1	6.0 ± 5.5	0.17	7.1 ± 4.8	5.5 ± 4.7	0.092
Operation time (min)	72.1 ± 16.8	106.6 ± 36.7	< 0.001	70.6 ± 16.7	105.9 ± 38.6	< 0.001
First defecation (d)	2.7 ± 1.0	2.5 ± 1.1	0.425	2.7 ± 1.0	2.6 ± 1.1	0.436
Full feeding (d)	8.1 ± 3.9	7.4 ± 2.6	0.213	8.4 ± 4.2	7.4 ± 2.4	0.126
Incision infection (case)	6	0	0.009	6	0	0.031
Length of stay (d)	14.2 ± 4.9	12.5 ± 3.4	0.004	14.6 ± 5.3	12.4 ± 3.0	0.011
Redo (case)	3	7	0.249	3	5	0.727

Chance of reoperation in open surgery was 4.8% and in laparoscopic procedure was 8.1%. The reoperation rates were similar (p > 0.05). The cause of reoperation was mainly adhesive intestinal obstruction in the open operation. In the laparoscopic group, there were two cases of reversion, which occurred 1 month after the operation, one case of adhesive intestinal obstruction (2 months after the operation), one case of missed diagnosis of jejunum septum (3 weeks after the operation), and one case of necrotizing enterocolitis (1 week after the operation). There was no statistical difference between the laparoscopic group and the open surgery group (p > 0.05).

## Discussion

Intestinal malrotation is a common cause of duodenal obstruction in neonates and infants. Malrotation is abnormal twisting and fixation of the intestine [1] which may cause torsion necrosis of the bowel, abdominal pain, and chyloascites [2]. Although laparoscopic Ladd’s procedure is controversial for newborns [3], the advantages of laparoscopic surgery are minimally invasive, aesthetic incision and less postoperative pain [4–6]. Torsion of the bowel will prolong the operative time and postoperative recovery [7]. Different ages may also recover differently. Therefore, we matched the PSM according to the sex, age, and degree of intraoperative torsion of the child to reduce the influence of these selection biases. A propensity matching study found that laparoscopy has an advantage in shortening the postoperative hospital stay, but the children in this study are older and the proportion of newborns in the study is small. In our study, most patients are newborns and all patients are below 1 year old.

This study confirmed that laparoscopy can decrease hospital stay and the chance of incision infection. However, we did not find a gap in the first bowel movement and adequate feeding after laparoscopy. Laparoscopic surgery will prolong the operation time, the transfer rate is low, and postoperative complications are similar to laparoscopic procedure except for incision infection.

In this study, children who underwent laparoscopic surgery had less time to stay and a lower rate of incision infection. Since appendectomy during surgery is a common practice in our center, incision infections also occur occasionally. The incision infection rate of the children in our study is similar to previous studies [3]. Incision infection requires long-term incision dressing and long-term hospitalization. The length of stay in our study is similar to previous studies [8]. The standards for discharge are usually different in different centers, resulting in different hospital stays. Our discharge standard is usually to be able to achieve adequate feeding and exclude other complications. Since laparoscopic surgery rarely causes infection of the incision, surgeons may be more inclined to discharge the child after adequate feeding.

The big series described conversion rates between 8 and 45% [9–11]. Although our center has more neonates with volvulus, our transit rate is 8.1% (5/62). Our research also confirmed that the laparoscopy method is also suitable for newborns and infants. During our procedure, we placed a laparoscopic trocar in the umbilical cord for observation, and 2 trocars are put in the right abdomen and right lower abdomen. This allows the surgeon to perform the operation comfortably. The procedure was similar to Pham reported [9]. Although Agrawal reported a “steering wheel” method to reset the twisting midgut [12], we found that for the most part the twisted intestine was not what they suggested [10]. We not only reset the bowel, but also probe the bowel from caecum to jejunum to ensure sufficient torsion reduction. We discovered that after the reduction of the torsion of the mesenteric root, some children had the folded part of the mesenteric torsion. So this exploration is very important, which may be the reason why our reversion rate is not high as reported. At the same time, in addition to the release of Ladd’s band, we also need to release the greater omentum from the transverse colon. Because of our two reversion cases, we found that both of the greater omentums were involved in the reversion of the bowel. Only sufficient release of the greater omentum can fully expand the mesenteric membrane. It has been reported that part of the intestine may retract after laparoscopic surgery, possibly due to inadequate release of the greater omentum. However, the majority of our patients with midgut volvulus underwent laparoscopic Ladd’s procedure successfully, and only two relapsed. This is different from what has been reported before. Even in the case of torsion, we attempted a laparoscopic reoperation without a recurrence. Although follow-up time was limited and the risk of reversal was lifelong, all of our cases recurred within 2 months after surgery.

Although a rapid postoperative recovery of peristalsis after laparoscopic surgery has been reported in many studies [8, 11, 13], we did not find that the laparoscopic group had a significant advantage in postoperative intestinal function recovery in our study as the other reports [14, 15]. Our method of judging the recovery of bowel function is by the time of the first defecation after surgery. The method of judging bowel function is not consistent, and it must be subjective, which may be the reason for the difference in results. Another reason for the difference in recovery may be the higher proportion of midgut volvulus in our study.

At the same time, we found that the operation time of laparoscopic surgery was longer than that of open surgery group. Many studies have the same opinion [13, 16, 17]. Our operation time is similar to the previous study [10]. This may be due to the high number of children with volvulus in our study.

As many reports have said, laparoscopic Ladd’s procedure has significantly higher difficulty in torsion and redo than open surgery. However, extended follow-up is necessary to determine the long-term efficacy of laparoscopic surgery. The incidence of postoperative adhesive obstruction after open Ladd’s surgery has been declared to be as high as 13%; Our incidence of postoperative adhesive intestinal obstruction is similar to about 10.5%. The unavoidable and extensive Ladd’s band separation during surgery may be the cause of postoperative intestinal adhesion obstruction. The treatment of asymptomatic dyspraxia remains controversial [3, 18].

Although the rate of reoperation was 8.1%, which is similar to of laparotomy as in the past literature [7, 19]. In the laparoscopic group, there were two cases of reversion, this may be due to the stenosis of the mesentery. At the same time, we found that the greater omentum enveloped the duodenum during the reoperation, so in addition to cutting the Ladd band, attention should be paid to the over-hyperplasia of the greater omentum. Although malrotation of the intestine with other intestine malformations is rare [20], it is difficult to detect it during surgery. Although there are many studies on necrotizing enterocolitis [21], the cause of necrotizing enterocolitis in our case is still unclear, intensive high frequency coagulation may be a reason [22]. It suggests that we need to pay attention to the abdominal signs during feeding. Complications seem to be unavoidable in both open and laparoscopic Ladd’s procedure with a certain chance of reoperation [19, 23], so it is important to inform the family members after discharge to avoid irreversible volvulus necrosis.

In summary, by a propensity score matching analysis, laparoscopic Ladd’s procedure is a safe approach even in infants and neonates. It can reduce the length of hospital stay and incision infection, but the operation time was extended, other complications are similar compared with open procedure for intestinal malrotation in neonates and infants. Therefore, it is recommended to be performed at the center skilled in laparoscopic technology.

## Data Availability

The data used for this manuscript can be provided for reasonable request to the editorial office after IRB approval.
